# Long Non-Coding RNAs in Psoriasis and Cutaneous Squamous Cell Carcinoma

**DOI:** 10.3390/jcm14145081

**Published:** 2025-07-17

**Authors:** Ioana Irina Trufin, Loredana Ungureanu, Salomea-Ruth Halmágyi, Adina Patricia Apostu, Simona Corina Șenilă

**Affiliations:** 1 Department of Dermatology, “Iuliu Hațieganu” University of Medicine and Pharmacy, 400006 Cluj-Napoca, Romania; ioana.irina.trufin@gmail.com (I.I.T.); h_salomea@yahoo.com (S.-R.H.); adinna.apostu@yahoo.com (A.P.A.); corina.senila@umfcluj.ro (S.C.Ș.); 2 Clinical Hospital of Infectious Diseases, 400000 Cluj-Napoca, Romania; 3 Department of Dermatology, Emergency County Hospital, 400006 Cluj-Napoca, Romania

**Keywords:** lncRNAs, psoriasis, cutaneous squamous cell carcinoma

## Abstract

**Background:** Long non-coding RNAs (lncRNAs) are increasingly recognized as pivotal regulators in both inflammatory and neoplastic skin disorders. Their implications in numerous biological processes, including gene expression, immune responses, and epidermal homeostasis, suggest potential applications as diagnostic and prognostic markers, as well as therapeutic targets. **Methods:** We conducted a literature search on lncRNAs involved in both psoriasis and cutaneous squamous cell carcinoma (cSCC), highlighting overlapping pathogenic mechanisms. **Results:** Several lncRNAs, such as *HOTAIR, MALAT-1*, *H19*, and *uc.291*, display dysregulated expression in both psoriasis and cSCC, influencing keratinocyte proliferation and apoptosis, immune modulation, cytokine signaling, and the synthesis of epidermal proteins. **Conclusions:** The intersection of lncRNA function in chronic inflammation and skin carcinogenesis underscores their role in mediating the transition from psoriatic inflammation to tumorigenesis, offering new insights into disease susceptibility; further investigation through functional studies and clinical validation are required. The study of lncRNA-mediated molecular pathways is particularly relevant given the increased risk of non-melanoma skin cancers and lymphoproliferative disorders among patients with chronic and severe forms of psoriasis.

## 1. Introduction

The skin, as the largest organ of the human body, is a protective barrier against multiple environmental challenges and pathogenic infections. The skin’s critical functions depend on the integrity of its structure; various signaling mechanisms control gene expression to maintain epidermal homeostasis. Although extensive research has investigated the role of coding genes, the regulatory functions of non-coding genes in skin biology remain to be thoroughly understood, uncovering actionable pathways [1,2].

Over the past two decades, numerous advancements in transcriptome sequencing and the GENCODE project have revealed that only about 2% of the genome encodes proteins, while the majority is responsible for encoding thousands of non-coding RNAs (ncRNAs) [3]. Based on the transcript length, they can be broadly classified into two categories: small non-coding RNAs (e.g., microRNAs) and long non-coding RNAs (lncRNAs), comprising molecules over 200 nucleotides in length [1].

Once dismissed as “transcriptional noise,” lncRNAs contribute to many biological processes, including epigenetics, transcription, posttranscriptional regulation, and protein translation. In the cytoplasm, lncRNAs can either promote or inhibit protein translation by binding to target mRNAs through base-pairing; moreover, they modulate signaling pathways by interacting with nucleic acids [2,4]. Nucleic lncRNAs can function as modifiers of chromatin accessibility, thus regulating transcription; in the cytoplasm, they influence the translation process [2]. Although there is no standard classification, lncRNAs can be characterized based on their location or their genomic transcription origin (Table 1) [1,2]. LncRNAs play pivotal roles in both physiological and pathological conditions, inflammatory disorders and autoimmunity, and tissue homeostasis and inflammation [2,5].

The exploration of their structure, expression, evolution, and functions in cell regulation is still in its infancy [3], with only about 200 lncRNAs functionally characterized of around 28,000 cataloged human lncRNAs [6]. Emerging evidence from microarray and high-throughput sequencing technologies suggests that they are critical not only for skin development but also for the development of cutaneous proliferative diseases, with implications in tumor progression and cellular proliferation, apoptosis, and differentiation [7].

## 2. Non-Coding RNAs in Keratinocyte Differentiation and Proliferation-Related Diseases

Under normal physiological conditions, the epidermis undergoes a monthly continuous renewal through a complex process, in which the normal function of basal keratinocytes needs to be maintained. The epidermal differentiation is possible through an optimal detachment of a subset of basal cells (progenitor cells) from the basement membrane and their migration outward through the epidermal layers. The dynamic balance between proliferation and differentiation is essential for epidermal homeostasis, which can be disrupted in a wide range of diseases, the most common ones being eczema, psoriasis, and keratinocyte-derived cancers [6].

Multiple studies have investigated the impact of lncRNAs on the integrity of both keratinocytes and immune cells. In their research, Kretz and co-authors demonstrated the importance of terminal differentiation-induced ncRNA (TINCR) as key regulator of human epidermal differentiation [8]. TINCR was described as a 3.7 kb nuclear and cytoplasmic intronic lncRNA that interacted with multiple differentiation-related mRNAs, facilitating their binding to the Staufen double-stranded RNA-binding protein 1 (STAU1), thus enhancing their stability in the upper epidermal layers [8].

PRANCR (progenitor renewal-associated non-coding RNA) was characterized as a novel lncRNA involved in epidermal progenitor cell replication, controlling the expression of 1136 genes involved in epidermal homeostasis [6]. PRANCR depletion in organotypic human epidermal tissue determined disruptions in the expression of genes encoding structural epidermal proteins, such as keratin 10 (KRT10), filaggrin (FLG), involucrin, and other proteins required for epidermal integrity; hence, PRANCR-deficient epidermis displayed altered stratification [6].

Conversely, anti-differentiation ncRNA (ANCR), an 855 bp cytoplasmic intergenic lncRNA, was shown to be downregulated in the process of terminal differentiation of keratinocytes [9]. Unlike TINCR or PRANCR, by further associating with the methyltransferase enhancer of zeste homolog 2 (EZH2), ANCR inhibited keratinocyte differentiation through a process that led to epigenetic silencing of target gene loci [9]. Additionally, DANCR (differentiation antagonizing non-protein coding RNA) was required for sustaining the undifferentiated phenotype of cutaneous progenitor cells; its expression was downregulated during terminal differentiation of keratinocytes [10].

Li C et al. demonstrated that lncRNA H19, a 2322 bp nuclear intergenic lncRNA, promoted keratinocyte differentiation by binding to miR-130b-3, thus reducing its activity and increasing the expression of desmoglein-1 (Dsg1), an important actor in epidermal stability [11]. Squamous cell carcinoma misregulated transcript-2 (SMRT-2) also influenced the differentiation process; its depletion resulted in the repression of a variety of genes engaged in epidermal homeostasis [10]. WAKMAR1 (wound and keratinocyte migration-associated lncRNA 1) was another lncRNA involved in the regulation of a network of protein-coding genes essential in cell migration [12].

In another study, the authors utilized microarray analysis to profile the impact of lncRNAs on keratinocyte differentiation, followed by validation of the results using qRT-PCR within a three-dimensional epidermal model of human keratinocytes. They showed that lncRNA AK022798 was upregulated in the early stages of differentiation, while lncRNA BC020554 was downregulated [13].

To conclude, lncRNAs exhibit both pro-differentiation and anti-differentiation roles through diverse mechanisms. However, further research is required to elucidate the regulatory implications of lncRNAs in specific biological functions.

In recent years, the existence of a link between dysfunctional lncRNAs and the development of various hyperproliferative skin disorders, including cutaneous squamous cell carcinoma, psoriasis, melanoma, hypertrophic scars, and hemangiomas, has been acknowledged.

## 3. Long Non-Coding RNAs in Psoriasis

Psoriasis is a chronic skin disease with an inflammatory and hyperproliferative background (epidermal and vascular hyperplasia, irregular differentiation of keratinocytes) affecting 1–3% of the general population [1,7]. An imbalance in skin immune functions is essential for the development of psoriasis, driven by interactions between dendritic cells (DCs), T cells, and keratinocytes (KCs). These lead to epidermal keratinization, lymphocyte infiltration, and extensive angiogenesis in affected skin lesions [7]. The abnormal activation of immunocytes, as well as the lymphocytic infiltration of mainly T cells, stimulates the expression of cytokines, thus determining an excessive proliferation of epidermal cells [4]. Psoriasis-related skin inflammation is frequently associated with systemic inflammatory manifestations, including metabolic or vascular disorders, as well as autoimmune and tumoral comorbidities [7].

Psoriasis has multifactorial determinants, among which genetic predisposition plays an important role; the 109 psoriasis susceptibility loci reported to date count for an estimated 60% of heritability [2]. Gene expression influences the development of psoriatic plaques; molecular keratinocyte-specific alterations are also found in non-lesional skin of psoriatic patients, creating a certain local sensitivity to the cytokines produced by skin-infiltrating lymphocytes [3].

Non-coding RNAs can modulate gene expression in various biological processes; recent data show that lncRNAs are involved in the development of psoriasis as critical epigenetic regulators [4]. Previous transcriptomic studies on psoriasis investigated mRNA expression levels in relation to their translated proteins, exploring their potential functions. This includes protein-coding genes linked to signaling pathways such as interleukin-17, Jak-STAT, or mitogen-activated protein kinase (MAPK) [5]. Although once considered transcriptional noise or junk, recent evidence demonstrated the implications of lncRNAs in psoriasis as emergent elements in both disease genesis and progression [5].

Different expression profiles of lncRNAs have been correlated with autoimmune and immune-related disorders, outlining a potential etiological connection that requires further analysis and in-depth interpretation [14]. To date, only a few lncRNAs associated with psoriasis have been investigated; they may function as inducers or silencers of keratinocyte proliferation via transcriptional regulation or by altering the expression of target mRNAs (Table 2) [5].

Psoriasis susceptibility-related RNA gene induced by stress (PRINS) was identified as one overly expressed transcript in uninvolved epidermic tissue of psoriatic patients and not in lesional psoriatic or healthy skin [4,15]. Széll et al. demonstrated this altered molecular network of non-lesional epidermal keratinocytes using organotypic skin cultures from both non-lesional and lesional skin areas of patients with psoriasis; their results proved the contribution of PRINS as a modifier in disease pathogenesis [3]. PRINS can influence the cellular stress response [3,4]; various trigger factors, such as UVB irradiation or viral infections, elevated PRINS RNA levels, creating a stress response in non-lesional keratinocytes [3,15].

Studies have shown that PRINS contributed to psoriasis susceptibility via regulation of G1P3 protein, previously identified in human neoplasms, with anti-apoptotic effects regulated by interferon-α [3,15]. In other words, the role of non-coding RNA PRINS in the pathogenesis of psoriatic lesions was related to the expression of G1P3 protein and a reduction in keratinocyte sensitivity to spontaneous apoptosis [3,4]. Moreover, quantitative real-time PCR and in situ hybridization (ISH) demonstrated an upregulated expression of PRINS-G1P3 up to 400-fold in non-lesional psoriatic epidermis, versus a 9-fold increase in healthy epidermis [15]. Szegedi and co-authors also reported decreased keratinocyte apoptosis in relation to dysregulated PRINS at the onset of psoriatic lesions [15]. Another study on the same problem identified a direct significant correlation between the G1P3 plasmatic expression levels and body mass index (*p* = 0.009) and an inverse significant correlation with age (*p* = 0.034) in 120 patients with psoriasis compared to 120 healthy volunteers using quantitative real-time PCR, suggesting the potential application of targeted therapy in a personalized medicine approach based on this specific axis [16].

The IL-23/IL-17 axis is of great importance in the pathogenesis of psoriasis, targeting the excessive proliferation and aberrant differentiation of keratinocytes, which further amplify the inflammatory process by secreting additional cytokines and chemokines in the local microenvironment [17]. LINC00958 was identified as an IL-17-induced long non-coding RNA overexpressed in cultured keratinocytes from psoriasis lesions compared to healthy skin samples; quantitative reverse transcriptase polymerase chain reaction (qRT-PCR) and single-molecule in situ hybridization confirmed its increased levels in lesional keratinocytes [17]. Moreover, the inhibition of LINC00958 determined a decline in IL-17-induced cellular proliferation (quantified by Ki-67 expression), highlighting the role of this lncRNA in the positive feedback circuit in psoriasis [17].

The interest in exploring the IL-23/IL-17 signaling axis helped researchers identify via RNA sequencing RP11-295G20.2 (later named CYDAER—cytoplasmatic differentiation-associated epidermal RNA), another IL-17A-induced lncRNA highly expressed in keratinocytes cultured from skin biopsies of psoriasis lesions [2]. The enriched CYDAER in the lower suprabasal epidermal layers contributed to their expansion, altering the path of keratinocyte differentiation in relation to IL-17A. Along with the uncovering of CYDAER, the authors also found overexpressed LINC01123 and LINC01215 in psoriasis keratinocytes, in line with previous findings regarding these lncRNAs [2]. Through computational analysis techniques using previously published RNA sequencing data from psoriatic and healthy patients, Stacey and Kõks demonstrated upregulation of LINC01215, pinpointing its contribution in the epithelial–mesenchymal transition (EMT) and the cell cycle pathway regulating psoriasis evolution [18].

The miR-424-5p/AKT/mTOR axis may also be a potential target for psoriasis therapy. Xian et al. detected via qRT-PCR and RNAscope^®^ in the skin tissue of psoriasis patients upregulated levels of AGAP2-AS1, a long non-coding RNA with implications in the biological mechanisms of psoriasis pathogenesis; it acts as a competitive endogenous RNA by sponging miR-424-5p to upregulate AKT3 and activate AKT/mTOR pathway, thus promoting cellular proliferation in keratinocytes [19]. In psoriatic tissue, qRT-PCR analysis found an overexpression of AKT1, positively regulated by BLACAT1 (the lncRNA bladder cancer-associated transcript 1), which also functioned as a competing endogenous RNA (ceRNA) in relation to miR-1495p [20]. On the other hand, the effects on the proliferation and inflammation of the AKT/mTOR axis were reduced by lncRNA-H19 in relation to the regulation of miR-776-3p using in vitro models [21].

Corresponding results were achieved following the exploration of MEG3 (lncRNA maternally expressed gene 3) in relation to the PI3K/AKT/mTOR signaling pathway; when activated, it could enhance inflammation while also inhibiting autophagy in psoriatic keratinocytes [22]. Previously demonstrated to be a regulator in different subtypes of cancer, the research extended the roles of lncRNA MEG3 to psoriasis pathogenesis and showed, using qRT-PCR and Western blot assays, that it suppressed inflammatory responses and facilitated autophagy via the inhibition of the PI3K/AKT/mTOR pathway in TNF-α-treated human keratinocytes and mouse psoriasis models [22]. Jia et al. also surveyed the role of MEG3 in psoriasis pathogenesis, applying qRT-PCR to measure the mRNA levels in a cell model for in vitro study; their findings were congruent: MEG3 inhibited cell proliferation and promoted apoptosis in activated cells by regulating miR-21 and suppressing caspase-8 expression [23]. One study focused on balancing the MEG3 and miR-21 expression with the corresponding tissular (psoriatic) levels of endoplasmic reticulum (ER) stress proteins such as GRP78, ATF6, and caspase-3, and identified, using ELISA (Enzyme-Linked Immunosorbent Assay), their low concentrations in psoriatic plaques compared to normal skin samples, in association with a low expression of MEG3 determined by qRT-PCR [24].

SPRR2C (small proline-rich protein 2C) was also involved in the PI3K/AKT/mTOR signaling pathway; highly expressed in a psoriatic cell model, SPRR2C regulated cell cycle proliferation and apoptosis while also inducing the expression of IL-1β, IL-6, IL-8, CXCL2, and CCL20, as demonstrated by qRT-PCR, FISH (Fluorescence In Situ Hybridization), CFSE (Carboxyfluorescein Succinimidyl Ester) proliferation assay, flow cytometry, Western blotting, and ELISA [25]. SPRR2C expression was significantly upregulated in psoriasis skin samples and in vitro cellular lines in response to IL-22 stimulation through the miR-330/STAT1/S100A7 axis [26]. The involvement of STAT3 signaling pathway in psoriasis pathogenesis displayed functions in inflammatory proliferation responses of keratinocytes and abnormal differentiation; FISH and qRT-PCR assays identified SPRR2G as a novel lncRNA that activated the STAT3 axis and was remarkably upregulated in psoriasis specimens [27]. Analogous research using an lncRNA microarray and qRT-PCR proved the implications of KLHDC7B-DT, an upregulated lncRNA that activated the STAT3 signaling pathway by inducing IL-6 and IL-8 secretion [28]. The IL-6 levels, along with other proinflammatory cytokines, were additionally influenced by the expression of lncRNA SH3PXD2A-AS1, involved in a positive feedback loop centering on STAT3 [29].

The JAK/STAT signaling pathway is critical for the pathogenesis of inflammatory and autoimmune diseases via the activation of cytokines and growth factors [30]. JAK/STAT disruption has been associated with psoriasis through the high expression of pathogenic mediators, such as Th1 cytokine interferon gamma (IFN-γ), contributing to the expression of genes involved in chronic inflammation [31,32]. The implication of lncRNAs in the JAK/STAT pathway was evaluated in a study published in 2021 conducted by Lin [31]. The authors identified 156 downregulated and 69 upregulated lncRNAs in a set of RNA-seq data from a psoriasis cohort and further evaluated potential lncRNA-PCG (protein coding gene) interactions, proving that PCGs associated with upregulated lncRNAs were enhanced in the IFN-γ-mediated signaling pathway [31]. Among the upregulated lncRNAs involved in this particular axis, PRKCQ-AS1, SH3PXD2A-AS1, and CERNA2 were correlated with STAT1, indicating their involvement in the pathogenesis and progression of psoriatic lesions through IFN-γ. Furthermore, the authors found an elevated expression of PRKCQ-AS1 and SH3PXD2A-AS1 in cultured cells stimulated by IFN-γ, validating the important role of this specific signaling axis [31].

In a study by Qiao M et al., the cytoplasmic lncRNA Msh homeobox 2 pseudogene 1 (MSX2P1) was significantly upregulated in psoriatic plaques compared to healthy skin biopsies, normal human epidermal keratinocytes, and human immortalized keratinocyte cells, based on immunohistochemistry, qRT-PCR, and Western blotting [33]. LncRNA MSX2P1 activated S100A7 by binding directly to miR-6731-5p and promoted the growth and progression of IL-22-stimulated keratinocytes [33]. The MSX2P1–miR-6731-5p–S100A7 pathway may represent a promising therapeutic target for future psoriasis treatment modalities [33]. Different in vitro research on S100A7 axis identified upregulated lncRNA MALAT-1 (Metastasis-Associated Lung Adenocarcinoma Transcript 1) as a regulator of keratinocyte proliferation, inflammation, and apoptosis, modulating IL-22-induced changes in cellular models [34]. There were also reports of genetic variants in MALAT-1 and psoriasis susceptibility evaluated in an Iranian cohort, which demonstrated a greater association of a specific SNP with the risk of psoriasis, opening questions about a potential risk locus for psoriasis in the Iranian population [35]. MALAT-1 is involved in diverse molecular mechanisms of both physiological and pathological dermatologic and immune conditions.

ANRIL (antisense non-coding RNA in the INK4 locus) was identified as a risk locus for psoriasis after the genotyping of four single-nucleotide polymorphisms (SNPs) in this lncRNA; specific haplotypes were more prevalent among psoriasis cases (lesional tissue samples form patients with psoriasis and age-/sex-matched controls enrolled in the study), with the recognition of allelic variants possessing protective roles and even genetic variables associated with the risk of cardiovascular disease [36]. ANRIL was proven to be involved in the epigenetic regulation of genes related to inflammatory, apoptotic processes and TNF-α secretion; the association with certain SNPs points to the multifactorial landscape of psoriasis [36]. The role of ANRIL was evaluated in a study that identified connections between genetic variants (six SNPs in ANRIL) and risk of psoriasis in northern China; specific genotypes of high and low risk were described in the studied population [37].

Similarly, the presence of SNPs modulated the expression or function of the HOX Transcript Antisense RNA (HOTAIR); three SNPs in HOTAIR were genotyped in 286 psoriasis patients and 300 control subjects, among which rs12836786 was a genomic variant significantly associated with the risk of psoriasis [38]. Yao et al. investigated this relationship in a Chinese Han population by screening three key candidate SNPs, including rs12826786, and found a statistically significant association with the risk of disease development [39].

Dysregulation of GAS5 (growth arrest-specific 5 lncRNA) was linked to autoimmune, cardiovascular, and neurological diseases, as well as cancers, in the framework of abnormal cell growth, proliferation, and survival [40]. Since GAS5 concentrations were controlled by degradation and not by modulation at a transcriptional level, its potential as a biomarker was associated with diabetes mellitus; evolving research using RT-PCR assessed the plasmatic levels of lncRNA GAS5 in patients with plaque psoriasis and revealed significant differences in the relative expression of GAS5 in psoriasis patients versus controls [40]. Moreover, the existence of a correlation between the plasmatic concentrations and psoriasis severity turns lncRNA GAS5 into a promising marker not only for diagnosing psoriasis but also for assessing the severity of the disease [40]. Similarly, serum lncRNA XIST was highly elevated in the serum of psoriasis patients and could differentiate them from healthy controls according to the receiver operating characteristic curve (ROC); in addition, higher levels of XIST were positively correlated with the PASI score and serum TNF-α, IL-17A, and IL-22 concentrations [41].

The growing repertoire of long non-coding RNAs as contributors to epidermal dysfunction in psoriasis and the discordant expression profiles in psoriatic tissue versus normal skin was validated in multiple studies. Zhi et al. disclosed via RNA sequencing and further validation by RT-qPCR 412 upregulated and 625 downregulated lncRNAs in lesional skin samples of psoriatic patients and their association with biological processes and pathways related to immunity, inflammation, and cell proliferation, including the MAPK signaling pathway [42]. Using qPCR and bioinformatic analysis, Wang and co-authors identified elevated levels of 48 lncRNAs, including lncRNA-AGXT2L1, in skin biopsies from psoriasis patients compared to healthy controls [4]. An overexpression of lncRNA-AGXT2L1 was also found in IL-17A stimulated keratinocytes, resulting in higher proliferation rates, decreased apoptosis, and a reduction in G0/G1 phase proportion; furthermore, co-expression network analysis indicated its interaction with estrogen-related receptor alpha (ERR-α) and codependency in stimulating keratinocyte proliferation [43].

LncRNA LOC285194 was also associated with the occurrence of psoriasis; its expression was reduced in skin lesion samples, and it was found to inhibit keratinocyte growth by sponging miR-616 in Western blot and qRT-PCR assays [44]. Moreover, keratinocyte exosomal LOC285194 was reduced in psoriasis lesions and had a negative relation with Th17 cell infiltration, leading to the inhibition of Th17 cell differentiation via the miR-211-5p/SIRT1 axis [45]. Psoriasis tissue samples also exhibited lower levels of MIR181A2HG, an lncRNA interacting with miR-223-3p and SOX6; the overexpression of MIR181A2HG inhibited the proliferation of keratinocytes in vitro, while its knockdown promoted cellular proliferation [46]. Matching results were obtained by Fan and colleagues, who performed qRT-PCR and Western blotting and also reported the downregulation of MIR181A2HG in psoriasis tissues, identifying 356 proteins to potentially interact with MIR181A2HG in keratinocyte proliferation [47].

Previously reported to affect cell proliferation in different cancers, MIR31HG was shown to be an upregulated lncRNA in psoriatic lesions compared to normal skin, in association with NF-κB, an important protein transcription factor; MIR31HG silencing inhibited the proliferation of keratinocytes while also prompting cell cycle arrest in the G2/M phase, as shown by flow cytometry analysis [48]. The NF-κB signaling pathway was activated by UCA1 (urothelial carcinoma-associated 1), another lncRNA highly expressed in psoriatic lesions, with implications in the enhancement of inflammatory responses, including cytokine signaling [49]. In a similar manner to the previously cited study, UCA1 silencing reduced inflammatory cytokine secretion and downregulated the expression of innate immunity-related genes in in vitro models.

Regulation of keratinocyte differentiation along with inflammatory responses were the most explored subject matters. Research showed that the uc.291 transcript influenced the activation of genes within the epidermal differentiation complex (EDC) [50]. Using RT-PCR and immunohistochemical studies, the authors demonstrated its downregulation in psoriatic lesions compared to non-lesional skin, pointing out its role in hyperproliferative skin disorders—not only psoriasis, but also poorly differentiated cutaneous squamous cell carcinomas [50].

**Table 2:** LncRNAs in psoriasis.

LncRNA	Molecular Targets	Expression	Function	References
PRINS	G1P3	Up	Hyperproliferation of keratinocytes via regulation of the anti-apoptotic G1P3	[3,4,15]
LINC00958	C/EBP-β and the p38 pathway	Up	IL-17-induced epidermal proliferation	[17]
CYDAER	Calcium-induced terminal differentiation	Up	IL17A-induced proliferation and altered differentiation of suprabasal epidermal layers	[2]
LINC01215	RUNX3 promoter methylation	Up	Promotes epithelial-mesenchymal transition	[2,18]
LINC01123	N/A	Up	Competing endogenous RNA for VEGFA	[2]
AGAP2-AS1	miR-424-5p/AKT/mTOR	Up	Activation of AKT/mTOR pathway by sponging miR-425-5p; promotes proliferation of keratinocytes	[19]
BLACAT1	miR-149-5p	Up	Increases epidermal thickness and inhibits apoptosis of keratinocytes by sponging miR-149-5p	[20]
SPRR2C	PI3K/AKT/mTOR miR-330/STAT1/S100A7	Up	IL-22-induced proliferation and inflammation	[25,26]
SPRR2G	S100A7 KHSRP (KH-type splicing regulatory protein)	Up	Regulation of proliferation, cell cycle, and apoptosis	[27]
KLHDC7B-DT	STAT3 and JNK signaling pathways by binding to ILF2 (interleukin enhancer binding factor 2)	Up	Promotes the proliferation of keratinocytes and induces the secretion of IL-6 and IL-8	[28]
SH3PXD2A-AS1	1. miR-125b/STAT3 2. JAK/STAT	1. Up 2. Up	1. Positive feedback loop stimulating proliferation of keratinocytes in psoriatic lesions 2. Autoimmune inflammatory responses	1. [29] 2. [31]
PRKCQ-AS1 CERNA2	lncRNA-miRNA-JAK/STAT pathway regulatory circuits	Up	Activation of autoimmune responses, hyper-inflammation in psoriatic skin	[31]
MSX2P1	miR-6731-5p–S100A7 pathway	Up	Promotes the growth and progression of IL-22-stimulated keratinocytes	[33]
MALAT-1	miR-330-5p/S100A7 axis	Up	Modulates IL-22-induced changes in relation to keratinocyte proliferation, inflammation, and apoptosis	[34,35]
ANRIL	ADIPOR1, VAM3, CASP14 genes	SNP polymorphisms	Modulation of immune response	[36]
HOTAIR	NF-κB activation	SNP polymorphisms	Mediates the expression of cytokines; regulates inflammatory responses	[39]
GAS5	GR protein	Up	Regulation of cell growth, proliferation, and cell survival	[40]
XIST	miR-338-5p/IL-6 axis	Up	Regulation of keratinocyte proliferation and inflammation; positively correlated with disease severity and inflammation	[41]
AGXT2L1	miR-484-mRNA axis	Up	Higher proliferation rates, decreased apoptosis, and reduction in G0/G1 phase proportion in IL-17A-stimulated keratinocytes	[4]
MIR31HG	NF-κB signaling pathway	Up	Promotes proliferation of keratinocytes	[48]
UCA1	NF-κB signaling pathway	Up	Enhancement in inflammatory responses, including cytokine production	[49]
uc.291	genes within the epidermal differentiation complex (EDC)	Down	Reduced expression of loricrin and filaggrin genes in lesional skin of psoriatic patients	[50]
MEG3	PI3K/AKT/mTOR miR-21	Down	Inhibits cell proliferation and promotes apoptosis via both the inhibition of PI3K/AKT/mTOR miR-21 signaling pathway and the regulation of miR-21 expression	[22,23]
H19	miR-776-3p	Down	Downregulation of H19 promotes the proliferation of keratinocytes and skin inflammation by upregulating miR-776-3p expression levels	[21]
LOC285194	miR-616 miR-211-5p/SIRT1	Down	Inhibits keratinocyte growth; inhibits Th17 cell differentiation	[44,45]
MIR181A2HG	miR-223-3p, SOX6	Down	Inhibits the proliferation of keratinocytes	[46]

## 4. Long Non-Coding RNAs in Cutaneous Squamous Cell Carcinoma

Aberrant proliferation of keratinocytes is the key element in cutaneous squamous cell carcinoma (cSCC) development. Associated with an important metastatic potential, significant morbidity, and mortality, cSCC is the second most common skin cancer, following basal cell carcinomas; it accounts for 20–50% of cutaneous neoplasms and is reported to have a rising incidence [51]. Ultraviolet radiation (UVR) exposure is the main etiopathogenetic risk factor, as well as age, immunosuppression, smoking, and genetic background [52]. UV radiation determines the accumulation of DNA damage in the epidermis, initiating cSCC carcinogenesis through uncontrolled proliferation of cells that carry the “UV signature” of the high mutational burden [52].

The mutational landscape of cSCC and the dynamic changes in tumor genomes pose a great challenge in long-term optimal treatment of advanced disease and are not fully elucidated. Next-Generation Sequencing (NGS) technologies have helped identify and measure different lncRNA transcripts in relation to several cellular processes, proving that their abnormal expression can lead to tumorigenesis [52]. However, their role in the cutaneous biology and pathogenesis of cSCC is still being explored; the upregulation or downregulation of lncRNAs can result in oncogenic or suppressive functions via the regulation of transcription factors, mRNA stability, or interaction with microRNAs (Table 3) [52].

Extracellular vesicles (EVs) are small vesicles connected to the cellular membrane that can be detected in bodily fluids; they are secreted by a wide range of human cells [51]. They are luminal or surface molecules with great potential in oncology and are attractive candidates for clinicopathological parameters such as cancer biomarkers or prognostic indicators [51]. Long non-coding RNAs are valuable components of EVs; using qRT-PCR, Wang et al. evaluated the serum of 30 cSCC patients and demonstrated high concentrations of lnc-PICSAR (p38-inhibited cutaneous squamous cell carcinoma-associated lncRNA (PICSAR), also named LINC00162) in EVs derived from cisplatin-resistant tumoral cells compared to healthy individuals and cisplatin-sensitive cells [53]. PICSAR contributed directly to cisplatin resistance by inhibiting miR-485-5p, which subsequently enhanced REV3L gene expression [53].

Dysfunctional cSCC-associated lncRNAs have been further investigated in various studies that provided evidence of their implications. LncPICSAR was upregulated in both primary and metastatic cSCC cell lines, promoting cutaneous carcinogenesis via the ERK1/2 signaling pathway (activation) and α2β1 and α5β1 integrin (downregulation), demonstrated by qPCR and flow cytometry analyses [54,55]. PICSAR was one of the earliest lncRNAs to be functionally characterized in cSCC; its expression was upregulated not only in cSCC tumor cells but also in actinic keratoses and cSCC in situ, pointing to its role in the early stages of cutaneous carcinogenesis [10]. Additionally, PICSAR’s contribution to tumoral progression and invasion is also related to its influence on cellular adhesion and migration by targeting integrin expression [10].

MALAT-1 has been proven to be a highly expressed lncRNA in many solid tumors; Zhang et al. explored its role in cSCC tumorigenesis, with qRT-PCR detection in cSCC cell lines demonstrating that MALAT-1 knockdown reduced cellular proliferation, migration, and increased apoptosis in relation to EGFR and the activation of MAPK and PI3K pathways; the authors reported elevated expression of MALAT-1 in relation to UVB stimulation [56]. In another study, qRT-PCR of 60 cSCC samples and 15 normal skin tissue samples showed that MALAT-1 also modulated the Wnt signaling pathway in cSCC, promoting proliferation and neoplastic migration [57].

The aberrant overexpression of HOTAIR leading to hyper-proliferating states was demonstrated in association with malignancy; the higher expression of HOTAIR in cSCC cells demonstrated by qRT-PCR assays enhanced their proliferation and migration rates, which were otherwise reduced by HOTAIR silencing [1,52]. Furthermore, higher expression of HOTAIR was directly correlated with lower survival rates in cSCC patients [58].

A tumor-promoting function was attributed to PRECSIT/LNC00346 (p53-regulated carcinoma-associated STAT3-activating long intergenic non-protein-coding transcript), HCP5 (human histocompatibility leukocyte antigen complex P5), H19, THOR (testis-associated highly conserved oncogenic), SCARNA2 (small Cajal body-specific RNA 2), PVT1 (plasmacytoma variant translocation 1), EZR-AS1 (ezrin antisense RNA1), and the long intergenic non-protein-coding RNAs LINC00319, LINC00094 (SERLOC), LINC00963, and LINC01048 [10,52,60,61,62,63,65]. These lncRNAs disrupt physiological processes of skin development and tissue regeneration, as validated by qRT-PCR studies using in vitro models.

Highly expressed in cSCC tumor cells in vivo, PRECSIT is associated with dysfunctional p53; loss of function in the p53 gene upregulated the expression of PRECSIT, which further regulated STAT3, activating MMP (matrix metalloproteinase) genes involved in cell migration and invasion [52,59]. The STAT3/VEGFR2 pathway was found to be affected by the overexpression of HCP5 in cSCC cells, an lncRNA that promoted malignant cellular behaviors and decreased apoptotic rates by competitively binding to miR-138-5p [60]. LncRNA H19 also interacted with p53; its high levels in both cSCC tissues and cSCC lines promoted the EMT (epithelial–mesenchymal transition) process and downregulated the expression levels of p53, thus promoting tumoral growth, migration, and invasion [61]. THOR contributed to cancer progression by interacting with IGF2BP1 (insulin-like growth factor 2 mRNA-binding protein 1); conversely, THOR silencing in cSCC cells induced the downregulation of IGF2BP1 mRNA and the inhibition of tumor progression [52]. SCARNA2 was previously identified in the development of colorectal cancer, but its expression was also upregulated in cSCC cell lines and was higher than in the adjacent non-tumor samples, results that suggest an oncogenic role and a potential therapeutic target [62]. The influence of overly expressed PVT1 in cSCC was evaluated using knockdown cell models; results showed that silencing PVT1 promoted apoptosis, suppressed cell proliferation, and inhibited the migration and invasion of cSCC cell lines, providing new insights into cSCC carcinogenesis [63]. Similar experimental models demonstrated the impact of EZR-AS1 on cSCC pathogenesis; its expression levels were significantly upregulated in cSCC samples and exhibited protumoral functions via regulating the PI3K/AKT signaling pathway [64].

Recent analyses revealed the implications of LINC00319 in cSCC; its upregulation promoted the expression of CDK3 (an important ATP-dependent serine/threonine kinase in controlling the cell cycle transition from the G0/G1 to G1/S phase) [52], as well as MMP-2, MMP-9, E-cadherin, and vimentin [10]. Evidence suggested LINC00319’s involvement in cSCC tumor size and lymphovascular invasion [10,52]. Elevated expression of LINC00094 was identified in cSCC cells and their metastases compared to normal skin samples, actinic keratoses, and cSCCs in situ, with higher expression levels in metastatic tissues compared to non-metastatic cSCCs, acting through regulation of MMP-1 and MMP-13 [65]. LINC00963 was also reported to be significantly upregulated in cSCC lines as well as in several other cancers; in cSCC, it regulates cell proliferation and migration via miR-1193/SOX4 axis (suppressing miR-1193 expression and promoting SOX4 upregulation and tumoral progression) [52,66]. LINC01048, upregulated in cSCC samples, was proven to be a poor prognostic factor in the evolution of the disease; patients with higher LINC01048 levels had lower overall survival and disease-free survival rates [10,52]. Studies showed that LINC01048 involvement in cutaneous cancer progression was via altering the Hippo pathway, leading to promoting the transcription of the YAP1 oncogene [10,52,67]. The clinical significance, prognostic value, and potential therapeutic biomarkers support the functional roles of these lncRNAs in keratinocyte cancer development.

TINCR (tissue differentiation-inducing non-protein-coding), an important lncRNA in epidermal differentiation, was downregulated in cSCC specimens compared to normal skin, as demonstrated by RNA interference in organotypic human epidermal tissue [8]. Research suggests that TINCR might play a role in ALA-PDT (5-aminolevulinic acid- photodynamic therapy)-induced effects on cSCC; the generation of reactive oxygen species (ROS) induced apoptosis and autophagy in tumoral cells via activation of the ERK1/2 pathway, which activated a transcription factor binding to TINCR and supporting its overexpression, thus promoting the inhibition of cSCC progression [68]. Both TINCR and SMRT-2 (squamous cell carcinoma misregulated transcript-2) stimulated the differentiation of keratinocytes, playing a protective role in carcinogenesis as potential tumor suppressors; however, their expression was significantly downregulated in cSCC cells [10]. The critical role of TINCR in epidermal stability was also related to Myc and TERC proto-oncogenes; TINCR determined methylation in the promoter regions of Myc and TERC genes, inhibiting the proliferation, migration, and invasiveness of tumoral cells, as demonstrated by overexpression techniques, methylation-specific PCR (MSP), and RNA immunoprecipitation (RIP) [69].

Auxiliary anti-proliferative activity in cSCC was attributed to the long intergenic non-protein-coding RNA 520 (LINC00520); studies show that LINC00520 mediated the inhibition of EGFR and further suppression of the PI3K/AKT signaling pathway and prevented tumoral progression, invasion, and migration of cSCC cells [52].

## 5. Potential Applications and Future Perspectives: LncRNAs at the Intersection of Psoriasis and Cutaneous cSCC Pathogeneses

With almost 70–98% of the cellular transcriptional output consisting of non-coding RNAs, the existing data on this topic remain fragmented, mostly due to the contrast between the vast and largely uncharted landscape of lncRNAs provided by microarray analyses and their yet undiscovered functions and regulatory roles in distinct tissues [70]. Present research endeavors focus on finding the clinical relevance and statistical significance of differentially expressed lncRNAs in specific pathologies. However, the inherent limitations of current studies, including small cohorts, heterogeneous methodologies, and the lack of longitudinal validation, contribute to the scattered nature of available findings.

As shown in the growing body of evidence, non-coding RNAs are important actors in numerous biological processes; their exploration not only helps understand the genetic framework of the disease but also contributes to identifying potential diagnostic biomarkers and new therapeutic options.

While there are no clinical trials currently focusing on using lncRNAs as a direct therapeutic target, a systematic review published in 2024 identified 14 completed, 7 ongoing, 1 suspended, and 1 clinical trial of unknown status investigating ncRNAs, including small-interfering RNAs (siRNAs), small-activating RNAs (saRNAs), and microRNAs (miRNAs) [71]. Although several studies were observational, the more recent ones were interventional phase 1 clinical trials investigating the therapeutic functions of specific ncRNAs in different solid and hematological neoplasia [71]. To date, clinical trials have been primarily limited to malignancies, with no registered trials currently available for non-neoplastic diseases. This highlights both the novelty of the topic and the need for broader research into the role of ncRNAs in other pathologies.

LncRNAs play a part in the genesis and progression of psoriasis. Additionally, as their expression seems to differ between pre-treatment psoriasis patients and healthy controls, lncRNAs may help predict the therapeutic response and course of the disease [72]. In their research, Fan and co-authors reported 10 immune-related lncRNAs as potential diagnostic biomarkers for psoriasis, with validated diagnostic efficacy (AUC > 0.7) [73]. They also recognized a connection between lncRNAs and treatment response (biological therapy), with statistically significant results that indicate their different expression in responder versus non-responder groups [73].

The exclusive expression of PICSAR by malignant keratinocytes in cSCC tissues, but not by normal keratinocytes in vivo, highlights their diagnostic purposes [55]. While cSCC is typically diagnosed through a full-body skin examination followed by a biopsy, this approach may require significant time or may not always be practical. Especially in high-risk cases, using EV-based liquid biopsy could enable earlier detection and reduce the need for unnecessary biopsies [53]. Moreover, elevated levels of PICSAR were demonstrated in EVs from cisplatin-resistant cSCC patients. The concentration of lnc-PICSAR in serum-derived EVs from cSCC patients could serve as a valuable indicator for guiding chemotherapy regimen selection [53].

Patients with severe forms of psoriasis and long-term disease have a higher risk of malignancy, among which non-melanoma skin cancers and lymphoproliferative neoplasia are the most common subtypes identified in this population [74]. Consequently, lncRNAs represent good candidates for the evaluation of this specific association; the diversity of molecular mechanisms they regulate require substantial efforts to identify which lncRNAs are at the meeting point of psoriasis and cSCC development.

In our research, we identified a subset of corresponding lncRNAs for both psoriasis and cSCC pathogeneses, as outlined in Table 4.

Research is slowly emerging, creating future novel directions for expanding current knowledge on the underlying molecular mechanisms in proliferative skin disorders. One relevant (previously mentioned) example is MIR31HG, an lncRNA with proven oncogenic properties in pancreatic ductal adenocarcinoma that is also involved in the hyperproliferative status of psoriasis lesions [75].

Finding additional convergent pathogenetic axes in inflammatory and malignant skin diseases helps highlight their important biological functions and clinical implications. For instance, the identification of common dysregulated lncRNAs in psoriasis and cSCC may be associated with defining susceptibility to either of the aforementioned conditions. LncRNAs can be valuable diagnostic tools and predictive markers for both the evolution of the disease and its therapeutic response. 

The incidence rates of skin cancers are rising worldwide [76]. Although a diagnosis of melanoma poses greater mortality, basal cell and squamous cell carcinomas are more prevalent [76], placing a significant burden on the healthcare system. Therefore, further exploration of molecular regulatory mechanisms in the development and progression of the disease, clinical biosignatures, and novel biomarkers and therapeutic targets are needed. Originating from epidermal stem cells, basal cell carcinoma (BCC) is the most frequently diagnosed tumor [70]; however, the role of lncRNAs in BCC pathology has not yet been validated. Sand et al. performed microarray analyses on RNA extracted from biopsies of 12 samples (6 BCCs and 6 controls) and identified a total of 1851 significantly upregulated and 2165 significantly downregulated lncRNAs [70]. The authors also pointed out the association of the genes encoding the most upregulated lncRNAs in BCC with lung adenocarcinoma, glioma cells, and a variety of other solid organ neoplasia, suggesting a potentially important role of differentially expressed lncRNAs in tumorigenesis [70].

Environmental carcinogens, such as UV radiation and UV-induced mutagenesis, as well as oxidative stress, provide a mutational landscape for skin carcinogenesis, with significant DNA damage carrying a “UV signature” [52,77].

Prior research has proven the implications of some non-coding RNAs (microRNAs) in the synthesis of melanin and melanoma development under UV stimulation, yet the role of lncRNAs in melanogenesis and melanocyte proliferation remains to be elucidated [1].

UCA1, previously described in relation to the enhancement in inflammatory processes in psoriatic tissue samples [49], is also differentially expressed in human melanocytes, with experimental data showing that UVB-induced melanin synthesis is downregulated with the upregulation of UCA1 [78]. Therefore, UCA1 expression is part of a negative feedback loop in UVB-induced melanogenesis and may be a potential target for the management of pigmentary skin disorders [78].

UV radiation is the main risk factor driving cSCC carcinogenesis; UVB rays specifically represent the most mutagenic and carcinogenic subclass in the solar spectrum, causing DNA photoproducts that further accumulate in the epidermal cells though their proliferation and differentiation [52,77].

The impact of UVB exposure on the expression of lncRNAs is yet to be uncovered. The specific expression of PICSAR in tumor cells, not only in cSCC but also in actinic keratoses, which are UV-induced premalignant skin lesions, is a promising direction of research; PICSAR might be further validated as a biomarker in the early diagnosis of cSCC [1]. In their study, Bernard et al. hypothesized that UVB irradiation induced alterations in the molecular structure of some ncRNAs [79]. Subsequently released into the surrounding microenvironment, they triggered an inflammatory response marked by increased production of tumor necrosis factor-α (TNF-α) and interleukin-6 (IL-6), thereby amplifying tissue damage and promoting the development of cancerous lesions [79].

UV-induced damage can therefore be attributed to the production of inflammatory mediators and reactive oxygen species (ROS), which accelerate cellular metabolism, favoring tumor progression, invasion, and metastasis, and facilitating the perpetuation of chronic inflammation [77]. This creates a vicious cycle with the augmentation in genomic oxidative damage [77]. Oxidative stress parameters in malignant tissue samples are elevated compared to their levels in the surrounding areas; these alterations can potentially create a distinct molecular profile of cSCC patients [77].

Chronic cutaneous inflammation and oxidative stress play an incontestable role in the pathogenesis of psoriasis, as highlighted by this systematic review including 79 original research papers on the significance of several oxidative stress markers in psoriasis and associated comorbidities [80]; although the value of both circulating and epidermal biomarkers has been established, the study of the genetic polymorphisms involved in the redox balance, including the novel interest in ncRNAs and their functions, creates promising perspectives.

Given the limited experimental data, additional research is needed to explore the role of lncRNAs in cutaneous pathophysiology and to evaluate their clinical significance. Such studies are essential to establishing the potential utility of lncRNAs in both the diagnosis and the management of proliferative skin disorders.

## Figures and Tables

**Table 1:** Classification of lncRNAs and their functions.

LncRNA Classification [Ref.]		Functional Roles [Ref.]
Based on their location [2]	Nuclear	Alteration of chromatin accessibility, regulation of transcription [2]
	Cytoplasmic	Control of RNA stability via the interaction with messenger RNAs, ribosomal RNAs, and regulatory RNAs [2]
Based on the genomic transcription origin [1]	Enhancer Promoter Intergenic Intronic Sense Antisense UTR overlapping	Regulation of gene expression (transcription, post-transcription, translation, post-translation), including epigenetic modifications [1]

Ref.—reference, UTR—untranslated region.

**Table 3:** LncRNAs in cutaneous squamous cell carcinoma.

LncRNA	Molecular Targets	Expression	Function	References
PICSAR/ LINC00162	miR-485-5p- REV3L ERK1/2 signaling pathway, α2β1 and α5β1 integrin	Up	Increases keratinocyte proliferation and migration, influencing cellular adhesion and migration by targeting integrin expression	[10,53,54,55]
MALAT-1	MAPK, PI3K, and Wnt pathways	Up	Increases tumoral progression and migration	[56,57]
HOTAIR	miR-326	Up	Enhances proliferation and migration rates; correlated with lower survival	[1,52,58]
PRECSIT/LNC00346	STAT3	Up	Increases cell migration and invasion via activation of MMP	[52,59]
HCP5	STAT3/VEGFR2, miR-138-5p	Up	Promotes malignant proliferation and decreased apoptotic rates	[60]
H19	p53, EMT process	Up	Involved in tumoral growth, migration, and invasion	[61]
THOR	IGFBP1	Up	Induces tumoral progression	[52]
SCARNA2	miR-342-3p	Up	Induces tumoral progression	[62]
PVT1	4EBP1	Up	Enhances tumoral proliferation, migration, and invasion; modulates apoptosis rates	[63]
EZR-AS1	PI3K/AKT	Up	Increases cell proliferation, migration, and invasion	[64]
LINC00319	miR-1207-5p CDK3, MMP-2, MMP-9, E-cadherin, vimentin	Up	Promotes cell proliferation and migration; also involved in tumor size and lymphovascular invasion	[10,52]
LINC00094	MMP-1 MMP-13	Up	Higher expression levels in metastatic tissues	[65]
LINC00963	miR-1193/SOX4	Up	Regulates cell proliferation and migration	[52,66]
LINC01048	TAF15 transcription factor, YAP1 via Hippo signaling pathway	Up	Poor prognostic factor; associated with lower overall survival rates	[10,52,67]
TINCR	ERK1/2 Myc TERC	Down	Induces apoptosis and autophagy in tumoral cells; stimulates the production of reactive oxygen species; inhibits the proliferation, migration, and invasiveness of tumoral cells	[68,69]
SMRT-2	not mentioned	Down	Stimulates the differentiation of keratinocytes; protective role in carcinogenesis	[10]
LINC00520	EGFR, PI3K-AKT pathway	Down	Prevents tumor progression	[52]

**Table 4:** LncRNAs in both psoriasis and cutaneous squamous cell carcinoma—expression and function.

LncRNA	HOTAIR	MALAT-1	H19	uc.291
Psoriasis	SNP polymorphisms; mediates the expression of cytokines; regulates inflammatory responses [39]	Upregulated; modulates IL-22-induced changes in relation to keratinocyte proliferation, inflammation, and apoptosis [34,35]	Downregulated; downregulation of H19 promotes the proliferation of keratinocytes and skin inflammation by upregulating miR-776–3p expression levels [21]	Downregulated; reduced expression of loricrin and filaggrin genes in lesional skin of psoriatic patients [50]
cSCC	Upregulated; enhances proliferation and migration rates; correlated with lower survival [1,52,58]	Upregulated; increases tumoral progression and migration [56,57]	Upregulated; involved in tumoral growth, migration, and invasion [61]	Downregulated, with consecutive downregulation of loricrin and LEC1C genes at both mRNA and protein levels [50]

## Data Availability

Not applicable.
